# A multi‐method evaluation of the implementation of a cancer teamwork assessment and feedback improvement programme (MDT‐FIT) across a large integrated cancer system

**DOI:** 10.1002/cam4.3719

**Published:** 2021-01-21

**Authors:** Cath Taylor, Jenny Harris, Karen Stenner, Nick Sevdalis, S A James Green

**Affiliations:** ^1^ School of Health Sciences Faculty of Health and Medical Sciences University of Surrey Guildford UK; ^2^ Centre for Implementation Science Health Service and Population Research Department King’s College London London UK; ^3^ Barts Health NHS Trust Urology Network Director Department of Urology Whipps Cross Hospital London UK

**Keywords:** health services research, healthcare quality improvement, implementation science, team training, teamwork

## Abstract

**Background:**

Globally, Multidisciplinary Teams (MDTs) are considered the gold standard for diagnosis and treatment of cancer and other conditions, but variability in performance has led to demand for improvement tools. MDT‐FIT (Multidisciplinary Team Feedback for Improving Teamwork) is an improvement programme developed iteratively with over 100 MDTs (≥1100 MDT‐members). Complex interventions are often adapted to context, but this is rarely evaluated. We conducted a prospective evaluation of the implementation of MDT‐FIT across an entire integrated care system (ICS).

**Methods:**

MDT‐FIT was implemented within all breast cancer MDTs across an ICS in England (*n* = 10 MDTs; 275 medical, nursing, and administrative members). ICS managers coordinated the implementation across the three stages of MDT‐FIT: set up; assessment (self‐report by team members plus independent observational assessment); team‐feedback and facilitated discussion to agree actions for improvement. Data were collected using process and systems logs, and interviews with a purposively selected range of participants. Analysis was theoretically grounded in evidence‐based frameworks for implementation strategies and outcomes.

**Results:**

All 10 MDTs participated in MDT‐FIT; 36 interviews were conducted. Data from systems and process logs covered a 9‐month period. Adaptations to MDT‐FIT by the ICS (e.g., coordination of team participation by ICS rather than individual hospitals; and reducing time protected for coordination) reduced Fidelity and Adoption of MDT‐FIT. However, the Acceptability, Appropriateness and Feasibility of MDT‐FIT remained high due to embedding implementation strategies in the development of MDT‐FIT (e.g., stakeholder engagement, interactive support).

**Conclusions:**

This is a unique and comprehensive evaluation of the multi‐site implementation of a complex team improvement programme. Findings support the imperative of considering implementation strategies when designing such programmes to minimize potentially negative impacts of adaptations in “real world” settings.

## Graphical abstract

This paper describes the prospective evaluation of the real world implementation of a cancer team improvement programme (MDT‐FIT) across an entire integrated care system, to inform the adaptation vs. fidelity debate in complex interventions. Although adaptations to the programme by the ICS reduced fidelity and adoption of MDT‐FIT, acceptability, appropriateness and feasibility were high due to embedding of implementation strategies (such as stakeholder engagement) in the development of the programme, and reinforce the imperative of such strategies in the design of complex programmes.

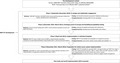

## 1 BACKGROUND

Globally multidisciplinary teams (MDTs) are considered the vehicle for delivering high quality, evidenced and equitable cancer care.^1^, ^2^, ^3^, ^4^ Regular multi‐disciplinary meetings (MDMs, also known as Multidisciplinary Case Conferences) are a mandatory part of cancer care services in many countries worldwide including the UK, Australia, and Canada, and there is growing evidence that these improve patient outcomes.^5^, ^6^, ^7^ However, there is evidence of variability in MDT performance and effectiveness.^8^, ^9^, ^10^ Accordingly, cancer policy in the UK^1^and internationally^11^, ^12^ recommends regular evaluation of MDT performance, and a range of tools have been developed for this purpose.^13^

**FIGURE 1:**
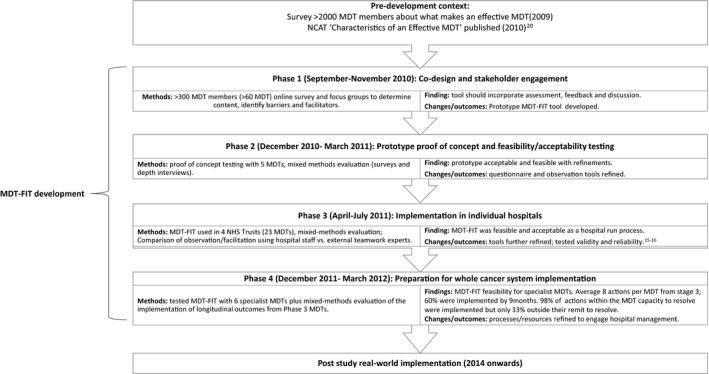
Summary of the development of MDT‐FIT

Multi‐Disciplinary Feedback for Improving Teamworking (MDT‐FIT) was a holistic team‐orientated improvement programme for UK cancer MDTs, co‐designed and tested with cancer MDT members from over 100 MDTs (Figure 1). MDT‐FIT comprises an assessment, feedback and improvement process, managed via an online platform. MDT‐FIT is intended and designed to be a developmental tool that empowers MDTs to determine their developmental needs and progress, rather than be used for performance management. This is important as ownership of team‐based interventions is known to impact on the level of team engagement and the implementation of improvements.^14^ Evaluation during development phases confirmed the feasibility and acceptability of the programme; that it did lead to implementation of improvements to teamworking; and confirmed the validity and reliability of integral tools used within the programme.^15^, ^16^ Implementation of MDT‐FIT in these developmental phases was, however, supported by health service researchers, and it had, therefore, not been implemented as intended: as a tool that is managed “in house” by hospital personnel.

This study reports the implementation and prospective evaluation of the MDT‐FIT programme across a large cancer integrated care system in England, by NHS hospital management and teams.

## 2 METHODS

### 2.1 Context/setting

This study took place across all breast cancer MDTs (*n* = 10) in an inner‐city integrated care system (ICS) serving a socially and ethnically diverse population of approximately 3.2million people in England. An ICS is *“a group of providers that come together in a formal*, *governed way to provide comprehensive*, *seamless cancer patient pathways*.*”* ^17^, ^18^ The ICS included 10 hospital Trusts, both local (district general hospitals) and cancer centres (specialist teaching hospitals). In the UK, core membership of MDTs is defined within Improving Outcomes Guidance^19^ and includes at least one of the following: breast surgeon, radiologist, histopathologist, breast care nurse, oncologist, and administrator. Individual MDTs could define their own membership for participation in MDT‐FIT beyond this core membership and thus some MDTs also included other clinical and non‐clinical members.

### 2.2 Intervention: MDT‐FIT programme

**FIGURE 2:**
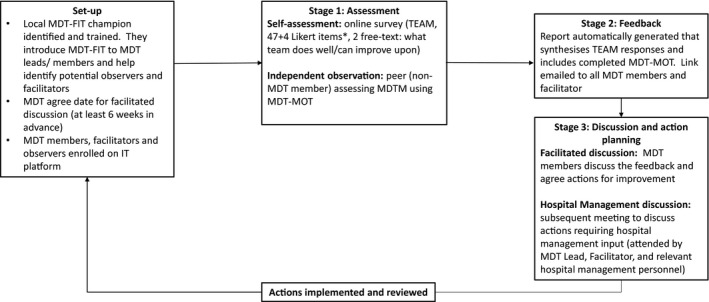
The MDT‐FIT improvement programme

MDT‐FIT is a teamwork assessment and feedback programme aimed at empowering MDT members to identify/diagnose and address issues in team performance. The tools encourage MDT members (and independent observers) to consider a wide range of team behavior and processes from leadership to clinical decision‐making and patient‐centredness, and infrastructure to governance, based on published recommendations for effective MDTs.^20^ The programme consists of three stages that take place over an 8–12‐week period: set up; assessment; and feedback followed by a facilitated discussion to agree actions for improvement (Figure 2). These stages are coordinated through an IT platform that includes a “dashboard” view for hospital managers and administrators to monitor progress. The programme includes all MDT members, together with members of hospital staff that undertake the roles of observer (to complete an independent assessment of performance in an observed MDT meeting); and facilitator (to facilitate the discussion and agreement of actions arising from feedback received from aggregate team member responses and observers report). Guidance regarding the appropriate background/skills for these roles is provided.

### 2.3 Implementation process

MDT‐FIT was designed to be coordinated by cancer managers/administrators within individual hospitals. For this implementation study this role was undertaken by an ICS administrator (non‐clinical), managed by the ICS Director. It was recommended that local hospital‐based “MDT‐FIT champions” be identified at each site for local support. The training package for the ICS administrator included an induction (e.g., comprising an introduction to MDT‐FIT and how it had been developed and tested to‐date, use of the IT system), and regular telephone support during the implementation provided by a health services researcher (JH). Comprehensive supporting materials were also provided on the MDT‐FIT platform (e.g., information sheets for different types of users; recommended specifications for skills/attributes of observers/facilitators; visual representations of the process; checklists; and step‐by‐step guides for using the IT platform etc.).

#### 2.3.1 Set‐up (weeks 1–4)

The ICS administrator was advised to visit each breast MDT (e.g., at their weekly MDT meeting) to introduce them to MDT‐FIT (a short presentation was provided for this purpose) and was also advised to identify a local “MDT‐FIT champion” that could help support engagement and coordination in each hospital. A fundamental logic rule built into the IT platform is that a team cannot move from the “set up” to “assessment” until a date for the facilitated team discussion has been entered and confirmed by the MDT Lead and facilitator. This is to ensure that all key stakeholders are engaged before the assessment stage starts. Once key steps had been completed (i.e., date for facilitated meeting entered and agreed by MDT Lead and Facilitator, and MDT Lead confirmed the team details are correct) the team automatically switched to the Assessment phase.

#### 2.3.2 Assessment stage (weeks 5–8)

Each MDT member received an automated invitation to log into the IT platform and complete the online assessment of their MDT (using TEAM^15^). Reminders were sent automatically to non‐responders 14 and 21 days after the initial invitation. The ICS administrator received weekly updates on response rates. Observers were sent the MDT‐MOT^16^ to assess team performance during observation of an MDT meeting; which they subsequently uploaded to the IT platform. The closing date for participation was automatically set at 2 weeks before the facilitated team discussion date but this could be extended by the ICS administrator to encourage further response if required.

#### 2.3.3 Feedback/discussion & actions stage (weeks 9–12)

2.3.3.1

###### 2.3.3.1.1 Generation of feedback reports

A PDF report is automatically generated and sent to MDT members and the facilitator. The report contains: a synthesis of TEAM responses (e.g., showing number of team members rating each aspect of teamwork as requiring improvement or not); and completed MDT‐MOT. The facilitator was able to access MDT members’ free‐text comments online and was provided with a proforma for recording actions arising from the facilitated team discussion.

###### 2.3.3.1.2 Facilitated discussion

The facilitated discussion was held, and the IT platform prompted facilitators to upload the completed Action Proforma (containing details of each agreed action including rationale, comments, barriers, aspect of teamwork [domain], person responsible and timeline for action, and date for review of progress). If a subsequent hospital management meeting was required, the ICS administrator was asked to arrange this, and the facilitator prompted to also upload the actions/timelines from this meeting.

### 2.4 Evaluation of implementation

We employed a theory‐driven multi‐method approach to evaluate the implementation of MDT‐FIT that included:

1. **Process and systems logs** – maintained by ICS administrator and researcher (JH). Study‐specific database used to record progress and issues for each MDT (summary of the issue/query, resolution or outcome, date received and resolved). All key dates for steps in implementation and on‐going issues were updated at least weekly. These dates were used to calculate the time taken by each MDT to complete each stage (Table 1). To enable real time resolution of the programming errors the IT platform was designed to capture details of usages (including time/date), any errors generated and process data (response rates to TEAM survey, completion of MDT‐MOT, and upload of actions). Any such IT or user errors, subsequent investigation and resolution was also logged.
2. **Interviews:** Semi‐structured telephone interviews were conducted with a range of purposively selected participants across all MDTs after participation, including MDT members, observers, facilitators, and ICS staff. Interviews were recorded with permission and sought to explore participants’ experience of MDT‐FIT including its strengths, weaknesses opportunities and barriers to use. Interviews were analysed thematically by two experienced qualitative researchers (CT/JH) independently before agreeing on a final coding framework that was applied across all data. Quotations are labelled with the role of the interviewee, except for those from ICS staff which are attributed collectively to preserve anonymity.

**TABLE 1:** Summary of timeframe for implementation of MDT‐FIT^a^ processes across the integrated cancer service

Month of implementation	MDT identifier										
	MDT 1		MDT 2	MDT 3	MDT 4	MDT 5	MDT 6	MDT 7	MDT 8	MDT 9	MDT 10
Set‐up Launch event											
1	OBS		OBS	OBS		OBS					
2					OBS		OBS		OBS	OBS	OBS
3											
4								OBS			
5											
6											
7											
TEAM^b^ response rate % (N/total N)	42 (5/12)		52 (12/23)	49 (19/39)	40 (4/10)	22 (14/63)	26 (10/38)	36 (5/14)	26 (5/19)	44 (12/27)	20 (6/30)
Key		=	Assessment phase commenced								
	OBS	=	Observation month								
		=	Feedback phase (facilitated discussion and action plan)								

^a^ Feedback for Improving Teamworking.

^b^ Team Evaluation and Assessment Measure.

The analyses of the data from these sources was theoretically grounded in evidence‐based frameworks for implementation outcomes and strategies. This analysis initially focused on identifying the extent to which implementation outcomes had been achieved based on mapping evidence from the above sources to Proctor's taxonomy of implementation outcomes.^21^ This involved mapping of evidence to six of the eight outcomes in Proctor's framework, namely: fidelity, acceptability, adoption, appropriateness, feasibility, and cost (Table 2). Penetration and sustainability were beyond the scope of this evaluation due to its time‐limited nature. The extent to which implementation outcomes were achieved was rated as being low, moderate, or high independently by at least two of the authors, and the evidence and ratings were discussed and agreed by all authors. Ratings were influenced by the extent and degree of evidence in each category: a “high” rating required substantial supporting evidence for the outcome; “moderate” required some supporting evidence but may be mixed in nature; and “low” ratings required little supporting evidence. To aid transparency, a supplementary file providing more of the evidence used to support ratings is provided. Alongside this process, evidence of implementation strategies that were used both in the development of MDT‐FIT and its implementation by the ICS were described in relation to their “fit” to the nine thematic clusters of strategies identified by Waltz et al^22^ (see Table 3). These nine clusters have 73 individual strategies within them; and the identified strategies were also mapped to these. For example, within the cluster “Use evaluative and iterative strategies” sits 10 strategies including “Assess for readiness/identify barriers and facilitators” which was one of the primary purposes of Phase 1 of MDT‐FIT development (see Figure 1), and “Audit and provide feedback” (which was a strategy used throughout all four phases of development of MDT‐FIT, Figure 1). The labels for these individual strategies and the source of their use is provided in Table 3. Finally, the data were explored for evidence of association between implementation strategies and outcomes (Table 3).

**TABLE 2:** The evaluation of MDT‐FIT^a^ implementation mapped to proctor's implementation outcomes^19^

Implementation outcome definition as applied to MDT‐FIT	Summary of key evidence (see also Table S1)	Rating
**Fidelity** Degree to which MDT‐FIT was implemented as intended by the program developers (e.g., in relation to adherence, dose and quality)	Implemented as intended: sourcing appropriately skilled facilitators and observers; completion of self‐assessment; observational assessment; and facilitated discussion. Some aspects supported by integral rules in the IT platform. Not implemented as intended: MDT‐FIT was coordinated centrally rather than hospital‐led; dedicated admin time for coordination was less than recommended (0.5 days/week rather than 2 days/week); No meetings with individual MDTs to inform and engage teams at set‐up were arranged; instead replaced by central launch event attended by members from 5/10 MDTs; Information sheets were not used consistently; ICS requested that MDTs shared their feedback reports; and Hospital management meetings (to address issues outside MDT’s capabilities to resolve) were replaced with an ICS‐wide event that had a different purpose.	Moderate
**Acceptability** Perception that MDT‐FIT (process and content and IT platform usability) is agreeable, palatable or satisfactory	Organisational level: Design as a cancer‐specific tool that was co‐designed with MDT members was valued; seen as useful for identifying common issues across the pathway that if addressed would improve patient outcomes. The minimal participation burden was acceptable, and the focus on practical solutions was beneficial. Team and individual level: Most interviewed team members (18/19) and facilitators (6/7) considered MDT‐FIT to be beneficial to team improvement. One team member felt usefulness was limited as his team was already high performing; and one facilitator felt that lack of team engagement impacted on its usefulness. Few issues were reported with the process or content of MDT‐FIT (work pressure cited as reason for non‐completion of the assessment; and value of observational assessment reliant on how typical the observed meeting was). IT Platform generally considered easy to use (5 observers; 13 team members; 5 facilitators). Feedback report and facilitated discussion were reported to have face validity and were useful and insightful. Observers and facilitators: useful for own professional development to observe an MDT outside their own MDT (6 observers, 4 facilitators). The MOT^c^ was said to be relevant and easy to use. However, poor preparation (poor understanding about expectations and roles) and procedural delays hampered the facilitation and observation process (3 observers, 3 facilitators).	High
**Adoption** Intention, initial decision or action to try MDT‐FIT	Organizational level: Influenced by alignment with organisational goals, vision, and existing processes Team and individual level: All 10 MDTs adopted MDT‐FIT and undertook all processes but their role in decision to adopt was not evident, some perceiving it to be mandatory. Individual team members could “opt out” by not completing the survey and/or not participating in the facilitated discussion.	Moderate
Appropriateness Perceived fit, relevance or compatibility of MDT‐FIT for the team/Trust/ICS, and/or perceived utility of MDT‐FIT to address issues/problems in teamwork	Organizational level: co‐designed with cancer MDTs and aligns with organizational goals to improve patient outcomes by improving team working. Perceived as not being too onerous and offered sustainable solution. Compatible with other leadership and development initiatives within the organization and viewed as adaptable to non‐cancer MDTs Team and individual level: MDT‐FIT was compatible with MDT goals to improve patient outcomes. Facilitators and observers found MDT‐FIT useful for own professional development as well as team working in their own teams. Some team members reserved judgment about benefits of MDT‐FIT and concerns were expressed by some MDT members about its potential misuse for performance management.	High
Cost (incremental or implementation cost) Cost impact of an implementation effort. Cost of delivering and implementing MDT‐FIT	Overall costs in terms of resources and time spent include: Cost of IT platform (server and IT support costs); and troubleshooting/support to coordination team Training of coordination team to use MDT‐FIT IT platform (approx. 2 hrs); then approx. 0.5 days/week for senior support/leadership; 2 days/week administrative support (depending on number of MDTs) MDT members: approx 1 hour 45 min (over 8–12 week period); MDT leads (1 additional hour), Facilitators (4 hours) and Observers (2.5 hours).	Costs were low
Feasibility Extent to which MDT‐FIT can be successfully used within ICS/individual Trusts	Feasibility of ICS use: MDT‐FIT can be ICS‐led if adequate resources and skills though local hospital‐based MDT‐FIT champions should also be included; There is a short learning curve during use with the first MDT for coordinators to understand process and systems: subsequent MDTs can be independently managed. Feasibility of recruitment, retention and participation: All 10 MDTs completed the process; Median completion time was 4 months (Table 1); Median TEAM^d^ survey response rate 38% (low survey response did not impede process as non‐responders contributed to facilitated discussion); Finding mutually convenient dates for facilitated discussions was challenging for some MDTs Feasibility of information support and IT system: All could access; mixed views on volume of information for facilitators and observers (3 facilitators finding this onerous, whereas others found it necessary and helpful); admin support for upload of observational report and actions proforma may be required. Minor bugs identified and remedied within 48 hrs to improve utility; most errors were user error (forgetting to add a team member) or sudden changes to the process (e.g., late changes to the facilitated meeting date), but overall the system was flexible enough to handle this, and this real‐world testing resulted in further refinements to the system and support materials	High

^a^ Feedback for Improving Teamworking.

^b^ Integrated Care System.

^c^ Meeting Observation Tool.

^d^ Team Evaluation and Assessment Measure.

**TABLE 3:** MDT‐FIT^a^ implementation strategies used and link to Implementation outcomes

Implementation strategy^20^	Strategies used in the development of MDT‐FIT (see Figure 1 for detail of phases)	Strategies used by ICS^b^ when implementing MDT‐FIT	Evidence of link to implementation outcome?
Use Evaluative and iterative Strategies	Assess for readiness/identify barriers and facilitators (Phase 1 MDT‐FIT); Audit and provide feedback (Phases 1–4); Purposefully re‐examine the implementation (Phases 2–4); Develop and implement tools for quality monitoring (IT system; Phase 4); implementation scale up (Current study); obtain and use stakeholder feedback (Phases 1–4); conduct cyclical small tests of change (Phases 2–4)	Limited evidence of this as was centrally implemented with little evidence of communication with MDTs except through the IT platform (e.g., inbuilt reminders). Held an end of implementation pathway meeting to obtain stakeholder feedback but unclear how/if this was used.	The cycles of testing and refinement meant that the final product was more likely to be acceptable (acceptability was high); assessment of barriers and facilitators to implementing MDT‐FIT was not carried out by ICS on an MDT level and contributed to the moderate adoption rating.
Provide interactive assistance	The MDT‐FIT development team provided facilitation and local technical assistance to MDTs when trialling the implementation of MDT‐FIT (Phases 2–4). The IT platform was designed to provide prompts and help users to navigate without need for further assistance.	The MDT‐FIT development team continued to provide centralized technical assistance to the ICS if required. Centralized technical assistance was also provided to MDTs by the ICS.	Related to feasibility (rated high) due to the assistance and prompts/user tested IT platform; All MDTs completed the process, participated in TEAM^c^, MDT‐MOT^d^ and agreed actions. Also related to the fidelity rating (moderate) where although much of the assistance was inbuilt to the system, there was a choice to have centralized assistance without local champions.
Adapt and tailor to context	During development (Phases 2–3) much adaptation and tailoring was enabled (e.g., content and method of assessment; type of observers; role of facilitators e.g., internal/external to the organization). From Phase 3, flexibility was supported regarding choice of observers and facilitators (evidence‐based guidance given) to enable adaptation to context and designed for single MDTs or whole systems MDT review. Inherent in the design (from Stage 1 onward) is that MDTs should have ownership of the assessment and determining their improvement priorities.	Centralization of coordination to ICS level was an adaptation; as were the decisions not to involve local champions or to have final “hospital” level meetings and instead to have a whole systems pathway meeting.	These were key adaptations that led to the moderate fidelity rating and to low “adoption” by some MDTs, however the negative impact of the adaptations was reduced by the high ratings of other outcomes such as acceptability and feasibility.
Develop stakeholder interrelationships	Stakeholder involvement was embedded from the start (Phase 1 consultation) and throughout the development of MDT‐FIT (Phases 1–4) where the priority was designing a tool that worked and would be used. The initial phases of development and testing involved identifying early adopters of MDT improvement (Phase 1), and identifying local champions is a recommended component of MDT‐FIT. MDT‐FIT was only tested with teams that had been adequately engaged and involved in the decision to participate (Phases 2–4) and testing across a broad range of MDTs was aimed at testing wider generalizability (Phases 3–4).	An ICS wide “launch” meeting was held but only 5/10 MDTs were represented. No local engagement events were held.	The high stakeholder involvement in the development of MDT‐FIT was associated with high appropriateness (fit with needs of teams and wide policy); high acceptability (content, method, and design was acceptable to the end user); and high feasibility. Also associated with the moderate fidelity and adoption ratings: the decision to control centrally and not to engage teams individually went against the premise of the tool being “developmental” rather than for performance management, and some teams reported the decision to participate felt mandatory rather than optional.
Train and educate stakeholders	Ongoing training for the coordinators of MDT‐FIT (local champions/cancer services manager) was provided throughout (Phases 2–4) and materials developed and adapted subject to stakeholder feedback for managers, facilitators, observers, and team members (Phases 3–4). Train the trainer strategies are inbuilt to the design, with the cancer services management team being trained initially (regarding MDT‐FIT as an intervention, and the use of the IT platform) and then their role is to engage the MDTs and train them.	Ongoing support was provided to the ICS coordinator; the centralized approach meant train‐the‐trainers was not implemented; some but not all training materials were distributed.	The use of these strategies in the development and implementation of MDT‐FIT was associated with feasibility: observers and facilitators could be found that were able and willing to perform those roles; and the feedback they provided was useful to teams. We would hypothesize that the lack of implementation of train‐ the‐trainers by the ICS would be associated with lower sustainability; but sustainability was not evaluated as part of this study.
Support clinicians	Clear instructions and reminders are integral to the IT platform (and were provided “manually” (e.g., via email) during earlier stages of MDT‐FIT development); it is a feedback tool designed to empower MDTs to take control of improvements; resources are shared on the IT platform.	This was integral to the IT platform; all staff had access to materials once they had registered. Some materials were not actively shared (or shared quite late into the process).	Linked to feasibility and the good participation rates.
Engage consumers	MDT members are the key “consumers” and over the four stages of development and testing of MDT‐FIT, over 1100 MDT members from approximately 100 MDTs have been involved in informing and/or testing MDT‐FIT; IT platform designed to ensure engagement up front (at set‐up) prior to being asked to participate.	A “launch” event was held with this purpose, but it was a centralized event and only 5/10 MDTs were represented; no local strategies were employed. Some staff reported feeling that participation was mandatory, rather than optional.	The reduced engagement strategies used during implementation are a key component of the moderate (rather than high) Fidelity rating, and also explain the moderate adoption rating following the lack of engagement with the key end users (MDT members).
Utilize financial strategies	NHS England funded the intervention development and were poised to plan roll‐out; designed to require minimal time and resources.	ICS invested by providing centralized staff time to manage the improvement project and coordinate participation, though other priorities resulted in the coordinators time for the project being reduced.	The ICS being unable to provide sufficient time for coordination was associated with the moderate fidelity rating (as shortcuts had to be made such as not visiting the teams in person, due to lack of time); and thereby to adoption at team level. The low cost of the intervention in relation to resource/time is a key factor in the appropriateness and acceptability ratings.
Change infrastructure	n/a	The ICS made participation of MDTs mandatory as it was designed to be used for pathway improvement.	The mandatory application (with limited stakeholder engagement) related to an adverse impact on adoption (rated moderate).

^a^ Feedback for Improving Teamworking.

^b^ Integrated Care System.

^c^ Team Evaluation and Assessment Measure.

^d^ Meeting Observation Tool.

## 3 RESULTS

All 10 breast cancer MDTs within the ICS participated in the implementation of MDT‐FIT, comprising a total of 275 medical, nursing, and administrative members. The teams ranged in size from having 10 (at a local general hospital) to 63 members (a specialist MDT that bought together several local teams using video‐conferencing). Although the launch event signified the start of the programme for all ten MDTs, the ICS took a staggered approach to starting the assessment phase after the launch event (with MDTs starting this within a 5‐month period). The time from starting assessment to the feedback phase varied from two to 6 months (five of the 10 teams completed the process within the recommended 3 months, whereas one other completed within 4 months; the others completed within 5 to 6 months Table 1).

A total of 36 interviews were completed, including 19 MDT members (at least one from each MDT: 11 were doctors, six of whom were MDT leads; five nurses; three MDT coordinators), seven observers, seven facilitators, and three key staff from the ICS.

### 3.1 Implementation outcomes

The findings as mapped to Proctor's implementation outcomes are detailed in Table 2 and discussed below. Fidelity is addressed first as we found issues with fidelity impacted on many of the other implementation outcomes.

#### 3.1.1 Fidelity *(rated moderate)*

Fidelity to the intended model of MDT‐FIT was rated moderate as the evidence showed that this was mixed: whilst some aspects were implemented with good fidelity, there were some key aspects of MDT‐FIT that were not implemented as intended (Table 2). These aspects were not mutually exclusive, for example, the central administrator having less than recommended time for supporting implementation meant that engagement with individual MDTs was not possible. Inadequate engagement coupled by delays in providing the information resources to staff, resulted in: a lack of understanding of the principles and purpose of MDT‐FIT and of their role within the process; some teams feeling obliged to participate rather than empowered to lead improvement; and contributed to delays in completing processes for some teams.

Furthermore, the choice by the ICS not to engage hospital based managers/administrators led to a staggered implementation of MDTs; this in turn meant that some MDTs had a long gap (up to 5 months) between being “engaged” at the central launch event and starting the process, during which time engagement waned (Table 1).

Despite these engagement issues, all teams completed the assessment and feedback stages of MDT‐FIT except that the ICS decided not to hold hospital management meetings to support MDTs to implement actions outside of their capability to resolve. Instead, all breast MDTs members were invited to share common learning across the ICS at a centrally run event. Whilst all teams were represented, this event focussed solely on sharing MDT issues (in an unstructured format) rather than on resolution plans. Finally, a key fidelity issue concerned an element built into the design of MDT‐FIT to prevent it being used for performance management purposes but rather as a supportive/developmental process. The feedback report is purposefully not shared with hospital management/administration to preserve anonymity and therefore encourage more honest (valid) appraisals. Instead, they can access the actions resulting from the facilitated discussion meeting, so they can support resolution of issues. However, interviews revealed that the ICS had requested access to the MDT feedback reports.

#### 3.1.2 Acceptability *(rated high)*

Most interviewees considered MDT‐FIT a useful tool for team development, and overall the different components were described as acceptable, and thereby acceptability was rated “high”. Team members found the self‐assessment component easy and quick to complete; observers found the observational assessment tool (MOT) acceptable to use; and MDT‐FIT instructions and IT platform were generally acceptable to all (Table 2). The exceptions to this were few but included concerns that its utility would be lesser for high performing teams.

Overall, the IT platform was stable and functioned well with only 13 reported issues all of which were resolved within a few days; in one case the website was blocked by the hospital firewall and this took over a week for local IT to resolve. Most problems related to human error by the senior administrator (*n* = 15) or where there were last minute changes (*n* = 5, e.g., to meeting dates) and/or where additional support or clarification was needed (*n* = 3). Such issues were used to inform real‐time improvements to the IT platform and support materials.

#### 3.1.3 Adoption *(rated moderate)*

Whilst ICS management made an active decision to “adopt” MDT‐FIT, this was not evident for individual MDT members and thereby this was rated “moderate.” Interviews with ICS management revealed that this decision was influenced by MDT‐FIT complementing existing tools and processes (within ICS and trusts) and the perceived fit with their organisational vision and the goal of improving patient care in cancer services. Feedback from some MDT members, however, indicated a lack of ownership of the decision to adopt MDT‐FIT due to the centralized management of implementation (Table 2). Although all 10 MDTs participated, this was perceived as mandatory by some, and often coupled with a lack of awareness of the potential benefits of participating. All teams had facilitated discussions and agreed action plans for improvement (Table 1).

#### 3.1.4 Appropriateness *(rated high)*

MDT‐FIT was considered to have good compatibility with individual, team, and organizational values and goals. For example, interviews with ICS staff supported appropriateness in relation to alignment with organizational goals, and that it could be applicable to non‐cancer services. Similarly, MDT members generally described MDT‐FIT as appropriate, beneficial and aligned with team and individual goals to improve patient outcomes; though some expressed suspicion about ICS using it for performance‐management, perhaps fuelled by the lack of fidelity (centralizing the coordination and poor engagement of MDTs) and adoption issues reported above.

#### 3.1.5 Costs *(rated low cost)*

Although a full economic evaluation for implementing MDT‐FIT was outside the remit for this study, overall costs in terms of resources and time spent were low (Table 2). Costs for implementation currently include training the individual(s) responsible for coordinating MDT‐FIT, and costs associated with access to the IT platform (e.g., server and support costs). Other costs include protected time in job plans for the administration at hospital and ICS levels; and the time of team members, observers and facilitators to participate (see Table 2). As improvement work is a requirement for cancer MDTs, the latter was absorbed as part of cancer services costs.

#### 3.1.6 Feasibility *(rated high)*

All ten MDTs completed the MDT‐FIT process, suggesting that feasibility for MDT‐FIT to be centrally coordinated was high. However, insufficient time allocation for coordination, and the decision not to appoint local MDT‐FIT champions impacted on both feasibility and effectiveness of the intervention. Despite these barriers, all 10 breast cancer MDTs participated; TEAM survey response rates ranged 20–52% (average 38% Table 1), and where low, the MDTs still saw value in the process and non‐responders participated in the facilitated discussion; and MDT‐MOT observations were completed in a timely manner (9/10 completed within 2 months from the start of assessment phase). Generally, once set‐up had commenced and MDTs entered the assessment phase, the process ran smoothly, and within expected timeframes for most MDTs (Table 1).

### 3.2 Implementation strategies and their relationship to outcomes

A wide range of strategies across eight of the nine clusters were used in the development of MDT‐FIT (change infrastructure not being relevant to the developmental phases, Table 3). In some areas, there was also evidence of similar strategies being used by the ICS in implementation (e.g., for the provision of interactive assistance) which related to the high feasibility rating. However, in other areas, the lack of explicit strategy at the ICS stage of implementation appeared to relate directly to poorer implementation outcomes. For example, the extensive use of evaluative and iterative strategies in the development of the MDT‐FIT Programme across all four phases of development, were associated with the high acceptability of the final product when implemented, but the reported lack of use of these strategies within implementation was associated with the moderate adoption rating.

Of particular interest is the relationship between adaptation and adoption. Adaptations to MDT‐FIT in the developmental phase were designed to ensure that the end‐product was “fit for purpose” and thereby to enhance adoption (as well as acceptability and other outcomes). However, the adaptations to MDT‐FIT during ICS implementation, particularly the centralization of implementation (which included the decision not to appoint local champions etc.) were key contributors to the low adoption by some MDTs, because it limited local ownership of the improvement process. There also appeared to be an “inverse” relationship between stakeholder inter‐relationships and change infrastructure: the poor stakeholder inter‐relationships (due to lack of engagement of individual team members as implementation was centralized) led to some team members perceiving that MDT‐FIT was mandatory rather than optional. This “mandatory” approach was also evident from the ICS who wanted MDT‐FIT to become part of the infrastructure (e.g., part of their strategy for cancer service improvement), however this approach was related to lesser rather than greater adoption of the improvement initiative by MDTs.

## 4 DISCUSSION

This study uniquely evaluated the implementation of a complex team improvement intervention within a “real world” large healthcare system using evidence‐based frameworks. Findings showed that it was possible to successfully implement the co‐designed team programme (MDT‐FIT) across a whole integrated care system, despite adaptations to delivery including centralizing its coordination. Implementation outcomes were only moderately impacted by this; appearing to be protected using key implementation strategies during the design and development of MDT‐FIT. The implementation success was determined by a combination of implementation strategies and decisions made in the “real world setting” and the implementation strategies embedded in the design and development of the programme.

Systems‐level interventions are rarely evaluated in relation to their implementation. The use of the evidence‐based frameworks for implementation strategies^22^ and outcomes^21^ in this study enabled an in‐depth exploration of both the programme development, and ICS implementation of the programme, on implementation outcomes. The findings showed that despite some aspects of fidelity and team‐level adoption being moderately implemented at best, other implementation outcomes were high (acceptability, appropriateness, and feasibility), and costs were low. The effective implementation of the programme in relation to these latter outcomes appeared to counter‐balance some of the less desirable outcomes (e.g., whilst MDT Leads/members did not feel they had a choice in adopting MDT‐FIT, they did perceive its usefulness/appropriateness and engaged in using it) and, thus, implementation overall was still successful.

There were novel and interesting findings regarding adaptation and adoption. There is a “fidelity vs. adaptation” debate in implementation literature based upon their assumed inverse relationship (you can only allow adaptation at the expense of fidelity) and increasing evidence of the need to allow contextual adaptation for an intervention to be acceptable across settings, and to enable sustainability.^23^, ^24^ Our evaluation indicated that whilst adaptation was fundamental to the design and development phases of MDT‐FIT, there were attempts to minimize the extent that it could be adapted when moving from the “research setting” to the “real world setting” (e.g., through rules built into the IT platform). On the whole, such strategies led to many aspects being implemented as intended, but adaptations made by the ICS to the process of implementation, particularly their centralized (rather than local) approach to this, served to enhance adoption at ICS level, but had an adverse impact on adoption at MDT level. Furthermore, the centralization of implementation constituted a “change infrastructure” strategy that made the programme integral to service improvement plans across the ICS, and thereby participation was perceived as “mandatory,” which also had an adverse impact on adoption at MDT level. Adaptations are rarely reported in adequate detail; it has been argued that their examination may help to identify which intervention components are “essential” to outcomes and which can be adapted and still produced the desired outcome within different delivery systems.^25^ MDT‐FIT was designed to be coordinated and supported at hospital level but was adapted to a centralized delivery model through an Integrated Care System. Whilst the adaptation did necessarily reduce fidelity, and impacted on adoption by teams, the implementation was successful in terms of other implementation outcomes due to the embedded strategies that deterred further adaptation. Use of MDT‐FIT at ICS systems level enables benchmarking between similar teams, with support and agreement of the individual MDTs at the outset. This may then confer further regional and national benefits, for example, by enabling the wider sharing of good practice.

The evaluation was not designed to assess the impact of the intervention on team outcomes (or patient outcomes related to team improvements). This would require an effectiveness/implementation hybrid study to determine the impact of adaptations on clinical/team outcomes, and to assess penetration and sustainability outcomes. Although these latter outcomes were not assessed as due to the time‐limited nature of the project, penetration within the pilot was evident as all 10 eligible cancer MDTs completed the process and representatives from all MDTs attended the centrally organized ICS event at the end of the process. Furthermore, outside the evaluation period, we received requests from two of the teams to resend copies of feedback reports for them to reflect upon progress and to inform future planning. Regarding sustainability, there were indications that it would be continued to be used by teams if supported by the ICS. Further the need for MDT improvement tools is supported in UK and global policy^1^, ^11^, ^12^ and despite delays to centralized national roll‐out in the UK, it has been used subsequently by MDTs across the UK since this evaluation.

The application of Proctor's framework was not without challenge, due to some aspects overlapping (e.g., the perceived usefulness and ease of practical delivery of MDT‐FIT could potentially be associated with both “Acceptability” and “Appropriateness” outcomes). Similarly, adoption outcomes could be further categorised into “conceptual” (e.g., reasons for adoption/non‐adoption) and practical (e.g., uptake and completion rates). It is accepted that measurement of implementation outcomes is underdeveloped.^26^ Further clarification of how the outcomes and strategies should be measured and applied in future studies would be beneficial in advancing understanding of their importance and role in implementation.

## 5 CONCLUSIONS

MDT‐FIT is a team improvement process designed to allow individual clinical MDTs to take ownership of their improvement goals, encouraging better team working, and improvements to patient care. This study has used evidence‐based implementation frameworks to provide a novel contribution to understanding about how such complex programme implementation might work in practice. Findings highlight the importance of using implementation strategies both in design/development phases and during implementation in order to enhance the likelihood of implementation success. MDT‐FIT is acceptable and feasible to implement. It has been designed to be coordinated/managed by hospital management, and therefore, if implemented on a larger scale (e.g., ICS) it is important that hospital‐based clinical and administrative champions are identified to ensure support and engagement at a local level.

## ETHICAL REVIEW

The protocol for this work was reviewed by UK National Research Ethics Service (NRES) and confirmed to be classified as service development and approved as such.

## Supporting information

Table S1

## Data Availability

Anonymised data are available on reasonable request.
