# Supplementary material for: A multi‐method evaluation of the implementation of a cancer teamwork assessment and feedback improvement programme (MDT‐FIT) across a large integrated cancer system
Source: Cancer Med. 2021 Jan 21;10(4):1240–52. doi: 10.1002/cam4.3719 (PMC7926008; doi:10.1002/cam4.3719)
Supplement: Supplementary file 1 — Table S1 [file CAM4-10-1240-s001.docx]

**Supplementary version of Table 2: The evaluation of MDT-FIT implementation mapped to Proctor’s implementation outcomes^19^**

| **Implementation Outcome**  ***Definition as applied to MDT-FIT*** | **Data source/s** | **Summary of key evidence from evaluation including illustrative quotes** |
| --- | --- | --- |
| **Fidelity**  *Degree to which MDT-FIT was implemented as intended by the program developers (e.g. in relation to adherence, dose and quality)* | Interviews  IT platform data | **Score: Moderate**  Most processes were implemented with fidelity to the intended model, including: sourcing appropriately skilled facilitators and observers; completion of self-assessment; observational assessment; and facilitated discussion. Fidelity was supported by integral rules in the IT platform, e.g. switch from set-up to assessment phase required confirmation from MDT lead and facilitator that a meeting date was confirmed. However, the following aspects of the MDT-FIT process were not implemented as intended:  **Coordination of MDT-FIT**   - MDT-FIT was coordinated from the head office of the ICS rather than hospital led administration (i.e. not via local MDT-FIT champions) - ICS coordinator had less dedicated time for MDT-FIT than recommended (estimated to be 0.1WTE instead of 0.4 FTE) - No meetings with individual MDTs to inform and engage teams at set-up were arranged; instead replaced by central launch event attended by members from 5/10 MDTs - Due to centralised coordination, recruitment of MDTs was staggered and thereby time from set-up to assessment varied (2-5 months, table 1)   **Use of resources**   - Information sheets designed to support individual roles were not consistently disseminated/used - ICS administrator bypassed IT platform rules system by accessing the system with the MDT-Leads login to agree provisional dates for meetings that had not been approved by the team.   **Hospital Management Meetings**   - No individual hospital management meetings were held (to address issues that were outside MDTs capabilities to resolve); instead an ICS wide event was held to ‘share learning’, but was not focussed on resolution plans as intended for these meetings. - ICS requested that MDT feedback reports were shared despite knowing that the reports were not shared with management as an integral design feature   Poor fidelity to the above processes resulted in: team members having limited understanding of the purpose and aims of MDT-FIT; inadequate preparation of team members, facilitators and observers to undertake and engage in MDT-FIT processes; and delays to the processes.  *“I felt that I wasn't engaged with the process until it actually happened (the meeting) even though I’m the lead”* (MDT lead)  *“It would have helped if the lead or someone had explained why it was important”* (MDT member) |
| **Acceptability**  *Perception that MDT-FIT (process and content and IT platform usability) is agreeable, palatable or satisfactory* | Interviews with MDT members, facilitators, observers and ICS staff about the general process/content of MDT-FIT, and about the IT system. | **Score: High**  18/19 team members and 6/7 facilitators considered MDT-FIT to be beneficial to team improvement. One team member felt it wasn’t beneficial as team was already functioning at a high level; and one facilitator stated that they felt that its usefulness was impacted by lack of team engagement. Few issues were reported with the process or content of MDT-FIT.  **Organisational level**  From the ICS perspective, MDT-FIT was acceptable as it had been developed as a cancer specific tool with input from MDT members. It was seen as useful for identifying common issues across the breast pathway that if addressed would improve patient outcomes. The minimal burden for MDT members in taking part was acceptable to ICS and the practical solutions the process enabled were also seen as beneficial.  *“It sits well alongside other things we were doing, like leadership and co-ordinator development… it’s diagnostic and should be an annual process… like a temperature check”* (ICS staff)  **Team and individual level**  MDT team members reported the self-assessment process as acceptable. Reasons for non-response were largely unknown though most non-responders did participate in the team discussion and some in interviews (citing that non-completion was due to work pressures). The opportunity to have a neutral observer to highlight ‘hidden’ aspects of team functioning was valued, although value depended on how typical the observed meeting was. The content of the feedback report and facilitated discussion were reported to have face validity and was useful and insightful.  *“It’s a useful process as it gives teams a benchmark and something to focus on”* (MDT member)  Observers and facilitators described their participation as useful for their own professional development and that they were able to reflect on their observations of other MDTs (6 observers, 4 facilitators). The Meeting Observation Tool (MOT) was said to be relevant and easy to use. However, poor preparation (poor understanding about expectations and roles) and procedural delays hampered the facilitation and observation process (3 observers, 3 facilitators).  **Acceptability of IT platform**  The IT platform was generally considered acceptable and ‘easy to use’ (5 observers/ 13 MDT members/5 facilitators); there were a small number of IT issues/bugs (Table 2) which were quickly resolved  *“It was fine, easy to register and complete the survey”* (MDT member)  Notable exceptions included the perception that MDT-FIT may be less useful/effective in high functioning teams (though these teams were still able to identify actions for improvement  “*It was not particularly useful but because we are a well-functioning team, but I can see it could be useful for more chaotic teams… the feedback report was useful and the facilitated MDT discussion interesting… and the meeting ended with clear action points”* (MDT member) |
| **Adoption**  *Intention, initial decision or action to try MDT-FIT* | Interviews with MDT members and ICS staff | **Score: Moderate**.  **Organisational level**  ICS decision to adopt MDT-FIT influenced by alignment with organisational goals, vision and existing processes  *“Human relationships in healthcare are the most important thing… and relates to the outcome patients receive… so it fitted well with our direction of travel to see the way clinical teams work together… we got involved because we thought it would help us to understand the variation in care... bigger Trusts may want to build MDT-FIT into their corporate process, link in with their transformation team so they can learn from the team’s actions”* (ICS staff)  **Team and individual level**  All 10 MDTs adopted MDT-FIT and undertook all processes but their role in decision to adopt was not evident, some perceiving it to be mandatory. Individual team members could ‘opt out’ by not completing the survey and/or not participating in the facilitated discussion.  *“It feels it was thrown at us, it felt we were told to take part rather than given a choice”* (MDT lead)  *“[Non-responders to the survey] were probably not bothered or just too busy … I think some couldn't be bothered, it wasn’t important to them”* (MDT member) |
| **Appropriateness**  *Perceived fit, relevance or compatibility of MDT-FIT for the team/Trust/ICS, and/or perceived utility of MDT-FIT to address issues/problems in teamwork* | Interviews with MDT members and ICS staff | **Score: High**  **Organisational level**  MDT-FIT had been co-designed with input from many cancer MDT members and aligns with organisational goals to improve patient outcomes by improving team working. It was compatible with other leadership and development initiatives in the Trust and ICS and viewed as adaptable to non-cancer MDTs  *“It sounded like a not too onerous process for team members… you can send people on team building but that’s not really sustainable... this tool offered a light touch and actions to build on”* (ICS Staff)  *“It was also appealing because it had been developed by Trusts and clinicians… was cancer specific… although think it could be applied to other clinical areas… it was innovative”* (ICS Staff)  **Team and individual level**  MDT-FIT was compatible with MDT goals to improve patient outcomes  *“It’s a useful process as it gives teams a benchmark and something to focus on”* (MDT member)  Facilitators and observers found MDT-FIT useful for own professional development as well as team working in their own teams.  *“It was interesting to see how another MDT runs compared to my own”* (Observer)  Some team members reserved judgement about benefits of MDT-FIT and concerns were expressed by some MDT members about its misuse for performance management. |
| **Cost (incremental or implementation cost)**  *Cost impact of an implementation effort. Cost of delivering and implementing MDT-FIT* | Cost of MDT-FIT (e.g. server/IT support costs)  Cost of time spent by coordinating team (ICS/hospital) and participation by team members | **Score: Costs were low**.  Overall costs in terms of resources and time spent include:   - Cost of IT platform (ongoing server and IT support costs); and central support to the hospital or ICS coordination team - Hospital-based coordination team (typically cancer services manager and deputies): training to use MDT-FIT IT platform (approx. 2hrs); then 0.1FTE for senior support/leadership; 0.4FTE administrative support for duration - MDT members: approx 1 hour 45 min; MDT leads (1 additional hour), Facilitators (4 hours) and Observers (2.5 hours). |
| **Feasibility**  *Extent to which MDT-FIT can be successfully used within ICC/individual Trusts* | Interviews  Data from IT platform | **Score: High**   - MDT-FIT can be ICS-led; however, it requires adequate resources and skills and should also include local MDT-FIT champions - Lack of familiarity/local knowledge of staff and their skills inhibits process, particularly identifying suitable observers/facilitators - There is a learning curve for coordinators to understand process and systems – after the first MDT has completed the process, subsequent MDTs can be independently managed.   **Recruitment, retention and participation**   - All 10 MDTs were recruited and completed the process - Median completion time was 4-months (table 1), ranging from 2 to 6 months and 5/10 completed within the suggested 3-month framework - Median TEAM survey response rate 38% (range 20 to 52%) - Low survey response did not impede process (non-responders contributed to team discussion) - Finding mutually convenient times/date to hold facilitated discussions was challenging for some MDTs   **Information support and IT system**   - All Trusts were able to access the system (one required their IT department to allow access); no problems were reported regarding individual team members accessing the system. - Mixed views on volume of information for facilitators and observers (3 facilitators finding this onerous, whereas others found it necessary and helpful. - All MDTs had an observational assessment; some observers and facilitators did not feel it was appropriate for them to have responsibility for data entry of their assessment/action proforma, instead stating that this should be an administrator’s task. - Minor bugs in IT system identified and remedied within 24-48 hours to enable more flexibility and utility, examples of such bugs included email invites not being sent when a participant was added after initial set-up, or reminders not being sent to some participants.   Most issues with the IT platform related to user error (forgetting to add a team member) or sudden changes to the process (e.g. late changes to the facilitated meeting date) (Table 2), but overall the system was flexible enough to handle this, and this real-world testing resulted in further refinements to the system and support materials  *‘For MOT there was clear guidance on what to rate and how, it was straightforward’* (observer)  *‘It was fine, easy to register and complete the survey’* (MDT member) |
